# A radiomics-based study of deep medullary veins in infants: Evaluation of neonatal brain injury with hypoxic-ischemic encephalopathy *via* susceptibility-weighted imaging

**DOI:** 10.3389/fnins.2022.1093499

**Published:** 2023-01-17

**Authors:** Xiamei Zhuang, Ke Jin, Junwei Li, Yan Yin, Xiao Dong, Huashan Lin

**Affiliations:** ^1^Department of Radiology, Hunan Children’s Hospital, Changsha, China; ^2^Department of Pharmaceutical Diagnosis, General Electric (GE) Healthcare, Changsha, China

**Keywords:** magnetic resonance imaging, deep medullary veins, hypoxic-ischemic encephalopathy, radiomics, neonatal

## Abstract

**Objective:**

The deep medullary veins (DMVs) can be evaluated using susceptibility-weighted imaging (SWI). This study aimed to apply radiomic analysis of the DMVs to evaluate brain injury in neonatal patients with hypoxic-ischemic encephalopathy (HIE) using SWI.

**Methods:**

This study included brain magnetic resonance imaging of 190 infants with HIE and 89 controls. All neonates were born at full-term (37+ weeks gestation). To include the DMVs in the regions of interest, manual drawings were performed. A Rad-score was constructed using least absolute shrinkage and selection operator (LASSO) regression to identify the optimal radiomic features. Nomograms were constructed by combining the Rad-score with a clinically independent factor. Receiver operating characteristic curve analysis was applied to evaluate the performance of the different models. Clinical utility was evaluated using a decision curve analysis.

**Results:**

The combined nomogram model incorporating the Rad-score and clinical independent predictors, was better in predicting HIE (in the training cohort, the area under the curve was 0.97, and in the validation cohort, it was 0.95) and the neurologic outcomes after hypoxic-ischemic (in the training cohort, the area under the curve was 0.91, and in the validation cohort, it was 0.88).

**Conclusion:**

Based on radiomic signatures and clinical indicators, we developed a combined nomogram model for evaluating neonatal brain injury associated with perinatal asphyxia.

## 1. Introduction

Susceptibility-weighted imaging (SWI) is a three-dimensional gradient-echo magnetic resonance (MR) imaging technique with high spatial resolution. The clinical importance of SWI in pediatric patients with neurological disorders has been reported for more than 20 years (Tong et al., 2007). SWI can provide additional information for the evaluation of various pediatric neurologic conditions, such as the detection of hemorrhagic lesions, better visualization of vascular malformations, cavernomas, telangiectasias, and Sturge–Weber Syndrome. SWI can also highlight the increased veins associated with venous stasis or oxygen extraction. The high sensitivity of SWI to oxygenated vs. deoxygenated hemoglobin magnetic susceptibility properties makes it useful in hypoxic and ischemic conditions (Chalian et al., 2011; Kitamura et al., 2011; Messina et al., 2013; Kim et al., 2020).

Brain injury in infants with hypoxic-ischemic encephalopathy (HIE) can impair neurological function (Zaghloul et al., 2020) and most often occurs in the central gray and white matter (WM) areas with the most vigorous metabolism (Chang et al., 2020). As a result of the hypoxic and/or ischemic injury, cellular energy metabolism is disrupted, ion pumps become dysfunctional, and glutamate accumulates in cells, leading to excitatory injuries. The oxygen extraction fraction is increased when the cerebral perfusion pressure is reduced, which can further increase the deoxy-to-oxyhemoglobin ratio within the venous blood that drains the injured brain areas. Prominent hypointense SWI veins may be associated with high deoxy-to-oxyhemoglobin ratios. Cerebral hypoxia and ischemia can lead to cerebral venous congestion and increased venous pressure, which in turn, can easily cause the deep cerebral and cortical veins to expand to varying degrees (Mukherjee et al., 2021). Neonates with hypoxic-ischemic brain injury and HIE have recently been reported to have abnormal, prominent veins (Messina et al., 2013), and there is a close relationship between the deep medullary veins (DMV) and WM injury in neonates (Arrigoni et al., 2011; Chen et al., 2020; Khalatbari et al., 2020; Benninger et al., 2021). In some infants, the veins are indeed prominent. In contrast, in others, this is not the case, and veins are not visible. Both scenarios are associated with a poor outcome (Kitamura et al., 2011). In infants, SWI abnormal findings are not only associated with sinovenous thrombosis, but also with WM lesions. Since the prominence of DMVs is associated with ischemic conditions, assessment is important.

Pathological changes can be observed in SWI, but few objective methods have been used to quantify its significance. Based on human perception alone, MRI provides limited information in various clinical situations. Radiomics involves extracting and analyzing features from digital medical images and converting them into data that could be analyzed (Huang et al., 2016; Sarioglu et al., 2022). Kitamura et al. (2011) used an SWI categorical grading scale to predict neurologic outcomes after hypoxic-ischemic injuries. In this study, we explored the potential role of DMVs in HIE-induced brain injury by developing and validating a combined nomogram model that integrates radiomic features with clinical characteristics.

## 2. Materials and methods

### 2.1. Patients and data collection

This retrospective study was approved by our institutional review board. The requirement for informed consent was waived due to the retrospective analysis of the anonymized data.

To identify neonates with perinatal asphyxia and HIE, the neonatology department database was analyzed between January 2018 and April 2022. We used the Queensland Clinical Guidelines to diagnose infants with HIE if they met all of the following criteria: (1) Apgar score≤5 at 5 and 10 min after birth; (2) profound metabolic or mixed acidosis with cord blood gas (pH <7.0 and/or base excess 12 mmol/L); (3) evidence of encephalopathy; and (4) multisystem organ failures, such as renal injury, hepatic injury, hematologic abnormalities, cardiac dysfunction, metabolic derangements, or gastrointestinal injury (Queensland Clinical Guidelines, 2021). The inclusion criteria were as follows: (1) Full-term neonates (gestational age >37 weeks) who underwent routine MRI scanning including SWI, at 5–10 days of life, and (2) demographic data, clinical information, and laboratory values were available. The exclusion criteria were as follows: (1) Premature infants (gestational age 37 weeks), (2) MRI scans with motion artifacts, (3) infants with metabolic disease, and (4) infants with massive intracranial hemorrhage. Sarnat scores are commonly used to evaluate the severity of neonatal HIE. Based on the clinical signs and electroencephalograms, according to the Sarnat criteria, HIE can be mild (stage 1), moderate (stage 2), or severe (stage 3) (Sarnat and Sarnat, 1976).

Neonates who underwent MRI scans within the first 2 weeks of life to investigate the possibility of congenital central nervous system malformations formed the control group. Infants without abnormalities observed on brain magnetic MRIs were included. The control group included 89 neonates with normal brain MRI, according to the radiologists’ consensus.

### 2.2. Image interpretation

For the patients in the HIE group, T1WI, T2WI, DWI, and SWI were retrospectively evaluated by two radiologists blinded to the clinical data. If the two radiologists disagreed, a third radiologist evaluated the images and his assessment was considered final. MRI abnormalities included abnormalities in the posterior limb of the internal capsule, basal ganglia, thalami, and WM.

### 2.3. Magnetic resonance imaging acquisition

All brain MRI scans were performed at our hospital using the 3.0 T MRI scanner (MAGNETOM Skyra, Siemens or MAGNETOM Prisma, Siemens) with an eight-channel head coil, set using the same MR parameters. The process is presented in detail in the Supplementary material.

### 2.4. Image preprocessing

Before segmentation and feature extraction, the images were preprocessed to remove potential differences between images that were acquired from the two different MR scanners. The details can be found in the Supplementary material.

### 2.5. Image segmentation and radiomic feature extraction

Susceptibility-weighted imaging images from each patient were independently reviewed by two pediatric radiologists (observer 1, a pediatric radiologist with 5 years of experience, and observer 2, a pediatric radiologist with 10 years of experience) who were blinded to the clinical data. Any discrepancy between the two radiologists was resolved by a third pediatric radiologist (a pediatric radiologist with 25 years of experience). In this study, the DMVs were assessed and quantified with the created regions of interest (ROIs) close to the lateral ventricles (Kim et al., 2020). ROIs were drawn for the right and left WM, involving the DMVs. We selected two axial slices to draw the ROI at the level where the DMVs showed a typical fan pattern of drainage into the subependymal vein (Supplementary Figure 1). When drawing the ROIs, we excluded the subependymal veins and the large cortical veins. Then, we used the AK software [Artificial Intelligence Kit v.3.3.0, general electric (GE) Healthcare] to extract the radiomic features. These included first-order, shape (shape), gray-level run-length matrix, gray-level co-occurrence matrix (GLCM), gray-level dependence matrix (GLDM), gray-level size zone matrix, and neighborhood gray difference matrix. The selected image transformations were wavelet transformations (wavelet). Level 1 = logarithmic transformation, parameter sigma selection 2.0 and 3.0, and local binary mode. Level 2 = radius 1.0 and subdivision select 1. In total, 1,316 features were identified.

### 2.6. Reproducibility

Radiomic reproducibility was evaluated by intra- and inter-observers. Two observers performed the ROI analysis. Sixty patients were randomly selected and delineated twice by observer 1 to ensure intra- and inter-observer reproducibility. The same procedure and delineation were conducted once by observer 2 to calculate the intra-observer intraclass correlation coefficient (ICC). An ICC 0.75 indicated good agreement. The remainder of the delineation was completed by observer 1.

### 2.7. Feature selection and model construction

First, using a ratio of 7:3, patients were randomly divided into two cohorts: Training vs. validation. Clinical features from univariate analyses (*p* <0.05) were included in the multivariate regression analysis. Features with *p* <0.05 in the multivariate regression analysis were included in the clinical model.

It is important to understand that some features contribute to the positive performance of classification, whereas others may add noise. We used the minimum redundancy maximum relevance (mRMR) approach to eliminate redundant and irrelevant features from our radiomic model. The least absolute shrinkage and selection operator (LASSO) was conducted to select effective and predictable features for high-dimensional low-sample-size data with collinearity problems. In addition to determining the number of features, features with non-zero coefficients were chosen based on a 10-fold cross-validation. The Rad-score was calculated as the sum of the selected features, weighted by the coefficients.

**FIGURE 1:**
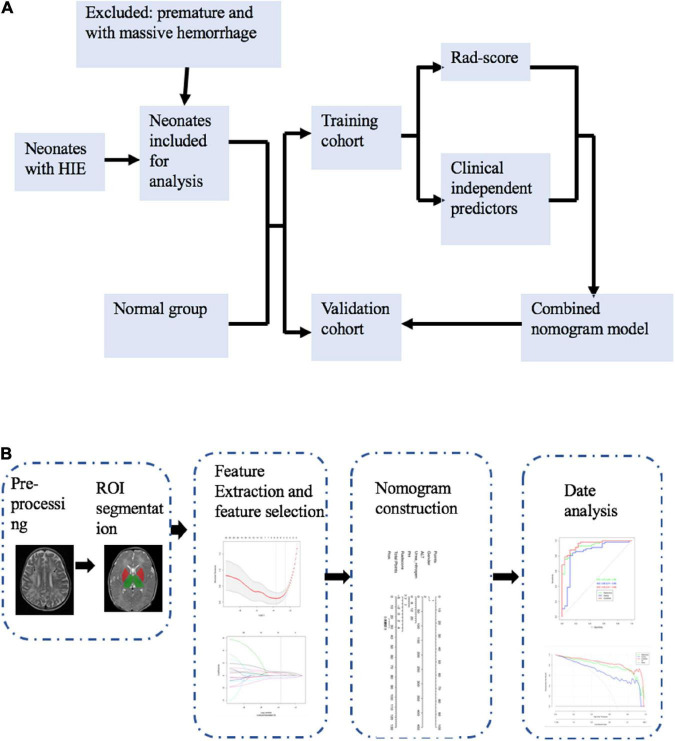
Study flowchart **(A)** and radiomics workflow **(B)**.

For the combined model, the clinical signatures from the clinical model and Rad-score were combined. Figure 1 illustrates the workflow of the radiomic analysis.

### 2.8. Model evaluation and validation

Based on the receiver operating characteristic (ROC) curve and area under the curve (AUC) analysis, the diagnostic efficacy of different models was analyzed in the training and validation cohorts. The Delong test was used to test the difference between the ROC curves. Different predictive models were calibrated and evaluated in the training and validation cohorts. Calibration curves were evaluated using the Hosmer–Lemeshow test. The decision curve analysis (DCA) was used to evaluate the clinical value of the different models.

### 2.9. Neurodevelopmental follow-up

Survivors underwent the Child Neurodevelopmental Psychological Development Table and Gesell Intelligence Test at 6, 12, and 18 months. The test was administered by a blinded psychologist. The outcome was dichotomized as “good” (development quotient scores>85) and “adverse” (death or development quotient scores≤85).

### 2.10. Statistical analysis

All statistical analyses were performed using SPSS software^1^ (Version 26.0) and R software^2^ (Version 4.1.0). Quantitative data were compared using Student’s *t*-test and Wilcoxon Rank test. Categorical data were compared using the χ^2^ test. For the analysis of the mRMR, the “mRMRe” package was used. We used the “glmnet” package to execute the LASSO and the “pROC” package to plot the ROC curves. Statistical significance was set at 0.05 for all two-sided tests.

## 3. Results

### 3.1. Clinical characteristics

This study included 279 neonates, including 24 with mild HIE, 166 with moderate-to-severe HIE, and 89 without HIE. According to the evaluation, 154 of the 190 HIE patients demonstrated a good outcome and 36 showed a poor outcome. At a ratio of 7:3, each patient was randomly assigned to either the training cohort or the validation cohort. Blood gas analysis was conducted at birth and further blood examinations were conducted upon admission to our hospital. The clinical characteristics and comparison between the HIE and normal groups are presented in Table 1. The clinical characteristics of the good vs. poor outcome groups are presented in Table 2. There was no statistical difference between the training and validation cohorts (*P* > 0.05).

**TABLE 1:** Comparison of demographic, clinical, laboratory features between control, and HIE groups.

Variable					Training cohort (*n* = 169)			Validation cohort (*n* = 83)		
	Total (*n* = 279)	Control (*n* = 190)	HIE (*n* = 89)	*P*-value	Control (*n* = 133)	HIE (*n* = 63)	*P*-value	Control (*n* = 57)	HIE (*n* = 26)	*P*-value
Birth weight (mean ± sd)	3.3 ± 0.5	3.3 ± 0.4	3.3 ± 0.5	0.689	3.3 ± 0.5	3.3 ± 0.5	0.441	3.3 ± 0.3	3.2 ± 0.5	0.579
Gestational time, week (mean ± sd)	39.3 ± 1.1	39.2 ± 1.1	39.4 ± 1.1	0.052	39.2 ± 1.2	39.5 ± 1.1	0.051	39.1 ± 1.0	39.3 ± 1.1	0.501
Age, day (mean ± sd)	8.7 ± 4.5	9.1 ± 4.2	8.5 ± 4.7	0.269	9.2 ± 4.3	8.7 ± 5.1	0.504	8.8 ± 4.1	7.8 ± 3.5	0.264
**Gender, *n*%**										
F	91 (32.6%)	41 (46.1%)	50 (26.3%)		30 (47.6%)	35 (26.3%)		11 (42.3%)	15 (26.3%)	
M	188 (67.4%)	48 (53.9%)	140 (73.7%)	0.002	33 (53.4%)	98 (73.7)	**0.005**	15 (57.7%)	42 (73.7%)	0.229
ALT (mean ± sd)	42.1 ± 62.0	18.6 ± 16.8	53.1 ± 71.7	**<0.001**	17.1 ± 9.7	52.8 ± 68.6	**<0.001**	22.2 ± 27.2	53.8 ± 78.9	0.047
AST (mean ± sd)	94.4 ± 966.0	44.7 ± 32.7	117.6 ± 106.6	**<0.001**	43.7 ± 26.1	116.7 ± 103.8	**<0.001**	47 ± 45.3	119.9 ± 113.8	**0.002**
Urea nitrogen (mean ± sd)	4.7 ± 3.0	3.4 ± 2.0	5.3 ± 3.2	**<0.001**	3.6 ± 2.1	5.3 ± 3.3	**<0.001**	3.0 ± 1.8	5.2 ± 2.9	**<0.001**
Creatinine (mean ± sd)	58.8 ± 34.3	38.9 ± 18.7	68.1 ± 36.0	**<0.001**	41 ± 18.1	67.7 ± 36.7	**<0.001**	33.9 ± 19.3	68.9 ± 34.6	**<0.001**
CK-MB (mean ± sd)	102.1 ± 108.6	44.3 ± 46.2	129.2 ± 211.3	**<0.001**	46.1 ± 49.2	130.6 ± 201.6	**0.001**	40.0 ± 38.4	125.9 ± 234.4	0.064
Procalcitonin (mean ± sd)	6.1 ± 14.6	1.8 ± 5.4	8.2 ± 17.0	**<0.001**	1.6 ± 3.6	9.5 ± 18.5	**0.001**	2.1 ± 8.3	5.3 ± 12.5	0.236
Lactic acid (mean ± sd)	5.5 ± 2.7	5 ± 1.2	5.8 ± 3.1	**0.012**	4.9 ± 1.3	5.8 ± 3.1	0.362	5.1 ± 0.9	6.0 ± 3.2	0.182
D-dimer (mean ± sd)	3.8 ± 6.7	2.3 ± 3.7	4.5 ± 7.6	**0.009**	2.3 ± 2.7	4.3 ± 7.2	0.027	2.5 ± 5.5	5.0 ± 8.7	0.175
CO2 (mean ± sd)	36.4 ± 12.9	34.8 ± 10.6	37.1 ± 13.8	0.177	36.2 ± 10.6	37.5 ± 14.2	0.541	31.5 ± 10.0	36.2 ± 12.8	0.100
PO2 (mean ± sd)	77.6 ± 35.3	73.4 ± 24.5	79.6 ± 39.2	0.171	70.8 ± 23.7	77.4 ± 30.6	0.130	79.6 ± 25.9	84.6 ± 54.3	0.656
HCO3-ion (mean ± sd)	21.3 ± 8.0	22.0 ± 4.9	21.0 ± 9.1	0.325	22.1 ± 5.3	21.1 ± 7.5	0.325	21.7 ± 4.0	20.8 ± 12.2	0.695
pH (mean ± sd)	7.2 ± 0.2	7.4 ± 0.1	6.9 ± 0.2	**<0.001**	7.4 ± 0.1	7.0 ± 0.2	**0.003**	7.5 ± 0.1	6.9 ± 0.2	**<0.001**
BE (mean ± sd)	−3.5 ± 7.9	−1.7 ± 4.5	−4.3 ± 9.0	**0.008**	−1.9 ± 4.9	−3.4 ± 8.6	0.211	−1.2 ± 3.0	−6.7 ± 9.3	**0.004**

The bold values represents that the *p*-value > 0.05, and the difference is statistically significant.

**TABLE 2:** Comparison of demographic, clinical, laboratory features between good, and poor outcome groups.

Variable					Training cohort (*n* = 134)			Validation cohort (*n* = 56)		
	Total (*n* = 190)	Good (*n* = 154)	Adverse (*n* = 36)	*P*-value	Good (*n* = 108)	Adverse (*n* = 26)	*P*-value	Good (*n* = 46)	Adverse (*n* = 10)	*P*-value
Birth weight (mean ±sd)	3.3 ±0.5	3.3 ±0.5	3.3 ±0.4	0.458	3.3 ±0.5	3.3 ±0.4	0.560	3.3 ±0.5	3.2 ±0.5	0.560
Gestational time, week (mean ±sd)	39.4 ±1.1	39.3 ±1.0	39.8 ±1.1	**0.008**	39.3 ±1.2	39.4 ±1.1	**0.022**	39.4 ±1.0	39.9 ±1.0	0.171
Age, day (mean ±sd)	9.8 ±5.9	8.5 ±4.7	8.2 ±4.3	0.060	8.1 ±3.9	9.2 ±5.6	0.224	8.3 ±5.2	11.2 ±6.6	0.129
**Gender, *n*%**										
F	50 (26.3%)	37 (24.0%)	13 (36.1%)		27 (25.0%)	10 (38.5%)		10 (21.7%)	3 (30.0%)	
M	140 (73.7%)	117 (76.0%)	23 (63.9%)	0.203	81 (75.0%)	16 (61.5)	0.257	36 (78.3%)	7 (70.0%)	0.883
ALT (mean ±sd)	53.1 ±71.7	46.4 ±64.2	53.1 ±71.7	**0.007**	48.1 ±69.6	98.8 ±103.9	**0.003**	42.3 ±49.7	37.6 ±25.6	0.771
AST (mean ±sd)	117.6 ±106.6	112.1 ±102.0	141.3 ±123.3	0.138	109.1 ±99.2	151.3 ±134.1	0.070	119.1 ±109.2	115.3 ±90.0	0.920
Urea nitrogen (mean ±sd)	5.3 ±3.2	4.8 ±2.5	7.0 ±4.8	**<0.001**	4.9 ±2.6	7.9 ±5.3	**<0.001**	4.7 ±2.3	4.8 ±2.2	0.876
Creatinine (mean ±sd)	68.1 ±36.0	66.1 ±32.1	77.1 ±48.9	0.096	66.4 ±35.3	86.3 ±52.3	**0.020**	65.3 ±23.4	53.2 ±28.5	0.153
CK-MB (mean ±sd)	129.4 ±211.3	128.4 ±200.8	133.5 ±254.6	0.896	123.1 ±202.7	121.1 ±220.2	0.966	140.9 ±197.8	165.7 ±340.2	0.755
Procalcitonin (mean ±sd)	8.3 ±16.9	7.0 ±14.7	13.9 ±23.8	**0.026**	7.0 ±15.2	14.2 ±21.9	0.050	6.9 ±13.6	13.3 ±29.5	0.289
Lactic acid (mean ±sd)	5.8 ±3.1	5.9 ±3.1	5.7 ±3.3	0.829	5.8 ±3.0	5.6 ±3.3	0.735	5.9 ±3.3	6.1 ±3.5	0.899
D-dimer (mean ±sd)	4.6 ±7.6	4.3 ±6.5	5.6 ±11.3	0.386	4.6 ±7.5	7.0 ±13.0	0.219	3.7 ±3.1	1.9 ±1.6	0.077
CO2 (mean ±sd)	37.1 ±13.8	36.7 ±13.0	38.6 ±17.0	0.466	35.6 ±12.4	36.2 ±11.6	0.824	39.4 ±13.9	44.9 ±26.3	0.348
PO2 (mean ±sd)	79.6 ±39.2	81.4 ±42.0	71.8 ±23.1	0.190	81.2 ±44.1	74.5 ±23.1	0.452	81.7 ±37.1	65.0 ±22.8	0.172
HCO3-ion (mean ±sd)	21.0 ±9.1	22.9 ±9.6	21.2 ±6.5	0.858	21.2 ±10.5	20.7 ±5.8	0.826	20.3 ±7.4	22.5 ±8.2	0.393
pH (mean ±sd)	6.9 ±0.2	7.0 ±0.2	6.9 ±0.2	0.736	7.0 ±0.2	6.9 ±0.2	0.914	6.9 ±0.2	6.9 ±0.2	0.705
BE (mean ±sd)	−4.3 ±9.0	−4.6 ±9.1	−3.4 ±8.6	0.504	−3.9 ±8.9	−3.8 ±7.6	0.929	−6 ±9.4	−2.6 ±11.2	0.318

The bold values represents that the *p*-value > 0.05, and the difference is statistically significant.

### 3.2. Univariate and multivariate regression analyses of the clinical characteristics

Multivariate regression analysis incorporated all parameters with a *p*-value of 0.05 from the univariate analyses. In the final analysis, sex, alanine aminotransferase (ALT) level, urea nitrogen level, and pH were identified as independent predictors of HIE (Table 3). The gestational time and ALT were identified as independent outcome predictors (Table 4). A clinical model was established using the independent predictors.

**TABLE 3:** Positive results of univariate and multivariate regression analysis between control and HIE groups.

	Univariate regression analysis			
Variable	Odds ration	Lower	Upper	*P*-value
Gender	0.393	0.210	0.736	0.004
Urea nitrogen	1.352	1.145	1.598	<0.001
CK-MB	1.012	1.005	1.019	<0.001
Procalcitonin	1.140	1.046	1.242	0.002
Lactic acid	1.158	1.005	1.335	0.043
D-dimer	1.131	1.010	1.268	0.034
pH	0.026	0.002	0.315	0.004
Creatinine	1.042	1.025	1.059	<0.001
ALT	1.074	1.042	1.108	<0.001
AST	1.032	1.019	1.045	<0.001
	**Multivariate regression analysis**			
**Variable**	**Odds ration**	**CI. 95**	***P*-value**	
Gender	0.2	0.08–0.49	<0.001	
ALT	1.07	1.02–1.11	<0.001	
Urea nitrogen	1.21	1.00–1.47	0.048	
pH	0.01	0.00–0.26	0.007	

**TABLE 4:** Positive results of univariate and multivariate regression analysis between good and poor outcome groups.

	Univariate regression analysis			
Variable	Odds ration	Lower	Upper	*P*-value
Gestational time	1.618	1.057	2.477	0.027
Urea nitrogen	1.247	1.095	1.420	<0.001
ALT	1.001	1.000	1.011	0.009
	**Multivariate regression analysis**			
**Variable**	**Odds ration**	**CI. 95**	***P*-value**	
Gestational time	2.46	1.34–4.51	0.004	
ALT	1.01	1.00–1.02	0.005	

### 3.3. Radiological features

Among the 190 patients with HIE, 38 had normal MRI findings and 152 had abnormal MRI findings. The median day the MRI was performed was on day seven of life. The patterns of injury seen were as follows. (1) Eighty-nine infants with injury to the cortex, subcortical and white matter had short T1 and short T2. (2) Punctate white matter lesions were seen in 52 infants. (3) Thirty-four infants had affected bilateral basal ganglia and thalamus with short T1. Bilateral DMV engorgement on SWI was seen on 65 of the 152 MRIs. Among the 65 patients, 14 had basal ganglia injury, 32 had watershed injury, and 19 had combined injury of basal ganglia and watershed.

Among the 190 patients with HIE, 95 patients received therapeutic hypothermia. From these patients, normal MRI scans were noted in 30 of the 95 infants.

### 3.4. Radiomic feature selection and construction of the rad-score

#### 3.4.1. The HIE group vs. the normal group

In distinguishing the HIE group from the normal group to build the differentiation model, all radiomic features with non-zero coefficients in the LASSO logistic regression model were selected (Supplementary Figure 2). From the 1,316 features in the training cohort, six potential predictors were selected after dimensionality reduction (Supplementary Figure 3). The Rad-score is a new radiomic signature developed using a specific equation (Supplementary Equation 1). Wilcoxon’s test was used to analyze differences between the groups. Supplementary Figure 3 shows the distribution of the Rad-scores for the training and validation cohorts. The HIE group had higher Rad-scores than the normal group in the training cohort (*p* < 0.001), which was confirmed in the validation cohort (*p* < 0.001).

#### 3.4.2. Good outcome group vs. poor outcome group

In distinguishing the good outcome group from the poor outcome group to build the differentiation model, all radiomic features with non-zero coefficients in the LASSO logistic regression model were selected (Supplementary Figure 2). From the 1,316 features in the training cohort, 14 potential predictors were selected after dimensionality reduction (Supplementary Figure 4). The Rad-score is a new radiomic signature developed using a specific equation (Supplementary Equation 2). Wilcoxon’s test was used to analyze differences between the groups. Supplementary Figure 4 shows the distribution of the Rad-scores for the training and validation cohorts. The poor outcome group had higher Rad-scores than the good outcome group in the training cohort (*p* < 0.001), which was confirmed in the validation cohort (*p* < 0.001).

### 3.5. Nomogram construction

**FIGURE 2:**
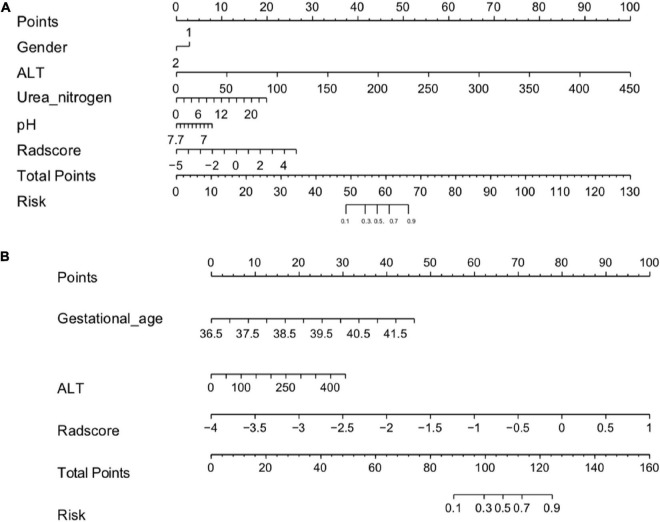
**(A)** Radiomics nomogram (HIE vs. normal). In the training cohort, the nomogram incorporated the Rad-score and gender, ALT, Urea nitrogen, pH. **(B)** A radiomics nomogram (good outcome vs. poor outcome). In the training cohort, the nomogram incorporated the gestational age, Rad-score and ALT.

Based on the results of the univariate and multivariate logistic regression analyses, the independent predictors of clinical characteristics were combined with the Rad-score to establish the nomogram (Figure 2 and Supplementary Equations 3, 4).

### 3.6. Performance and validation of different prediction models

#### 3.6.1. Hypoxic-ischemic encephalopathy group vs. normal group

**FIGURE 3:**
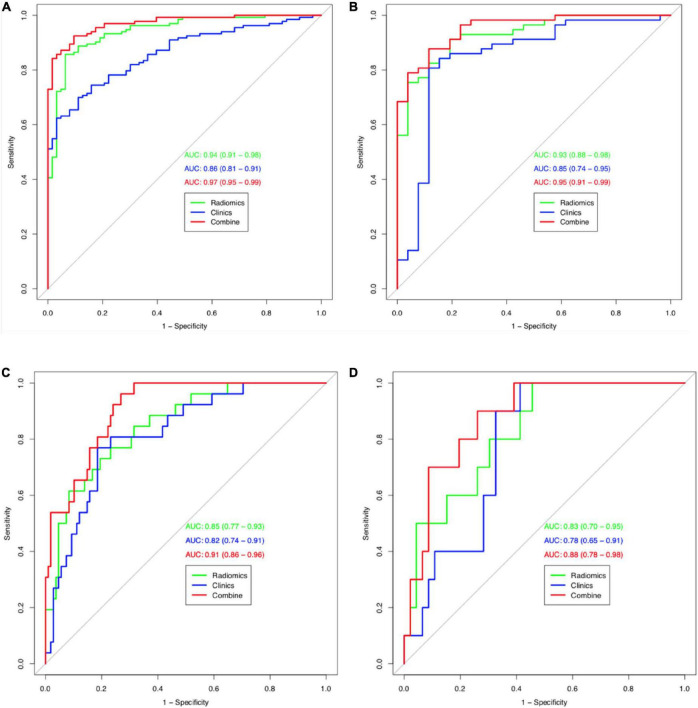
Receiver operating characteristic (ROC) curves (HIE vs. normal) for training cohort **(A)** and validation cohort **(B)** for tree models. Receiver operating characteristic (ROC) curves (good outcome vs. poor outcome) for training cohort **(C)** and validation cohort **(D)** for tree models.

**FIGURE 4:**
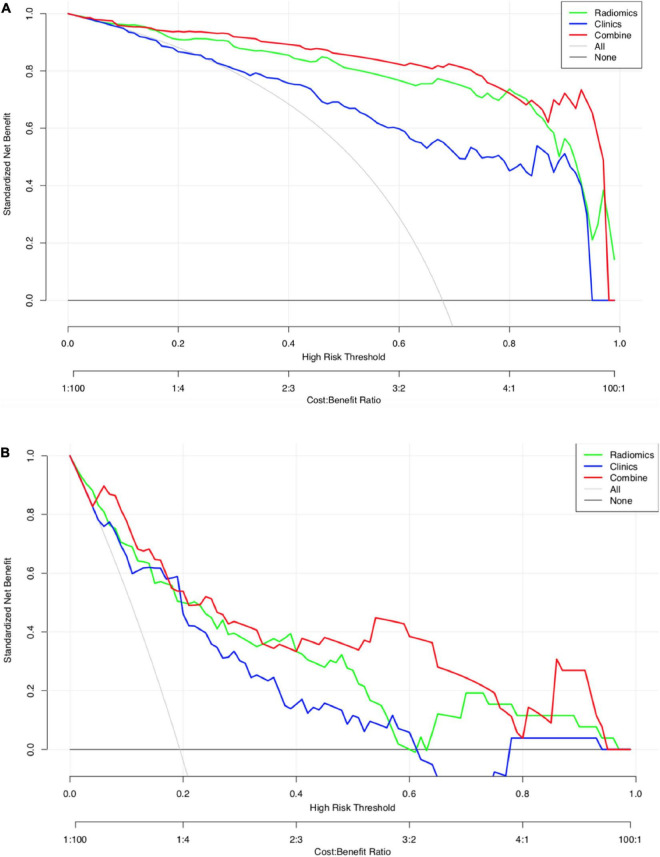
**(A)** Decision curve analysis for the three models (HIE vs. normal). The green line and red line represent the radiomics model and combined nomogram model. The blue line represents the clinical model. Decision curves showed that combined nomogram model achieved more clinical utility than radiomics model and clinical model. **(B)** Decision curve analysis for the three models (good outcome vs. poor outcome). The green line and red line represent the radiomics model and combined nomogram.

The calibration curves for the clinical, radiomic, and combined models showed good agreement with the observed values (Supplementary Figure 5). According to the training cohort, the AUC of the clinical, radiomic, and combined nomogram models were 0.86, 0.94, and 0.97, respectively. According to the validation cohort, the AUC of the clinical, radiomic, and combined nomogram models were 0.85, 0.93, and 0.95, respectively. The following sets of ROCs comparisons were all significant. The radiomic model compared with the clinical model (*p*-value = 0.009762), the radiomic model compared with the combined model (*p*-value = 0.02457), and the clinical model compared with the combined model (*p*-value 0.0001) in the training cohort. The ROCs of the clinical and combined models also differed significantly in the validation cohort (*p* = 0.01834). No significant differences in the ROC were found between the radiomic and clinical models or between the radiomic and combined models in the validation cohort (*p* = 0.1792). The combined model also showed the highest accuracy (accuracy: 0.918, sensitivity: 0.925, specificity: 0.905, PPV: 0.953, and NPV: 0.851) (Table 5 and Figure 3). The DCA based on these three models is shown in Figure 4. The decision curve showed that using the combined model to predict HIE provided greater benefits than when using the clinical and radiomic models.

**TABLE 5:** Accuracy and predictive value between three models.

	AUC	Accuracy	95% CI	Sensitivity	Specificity	PPV	NPV
**HIE vs. normal**							
**Training cohort**							
Clinical model	0.86	0.735	0.667–0.795	0.624	0.968	0.976	0.550
Radiomics model	0.94	0.883	0.829–0.924	0.857	0.936	0.966	0.756
Combined model	0.97	0.918	0.871–0.953	0.925	0.905	0.953	0.851
**validation cohort**							
Clinical model	0.85	0.699	0.588–0.795	0.614	0.885	0.921	0.511
Radiomics model	0.93	0.831	0.733–0.905	0.807	0.885	0.939	0.676
Combined model	0.95	0.867	0.775–0.932	0.877	0.846	0.926	0.759
**Good vs. poor outcome**							
**Training cohort**							
Clinical model	0.82	0.805	0.728–0.869	0.769	0.815	0.500	0.936
Radiomics model	0.85	0.769	0.687–0.837	0.769	0.768	0.444	0.932
Combined model	0.91	0.776	0.696–0.843	0.962	0.731	0.463	0.988
**Validation cohort**							
Clinical model	0.78	0.679	0.540–0.797	0.500	0.717	0.278	0.868
Radiomics model	0.83	0.732	0.596–0.841	0.600	0.760	0.353	0.897
Combined model	0.88	0.786	0.656–0.884	0.800	0.783	0.444	0.947

#### 3.6.2. Good outcome group vs. poor outcome group

The calibration curves for the clinical, radiomic, and combined models showed good agreement with the observed values (Supplementary Figure 6). The combined model showed the highest accuracy (Table 5 and Figure 3). The DCA based on these three models is shown in Figure 4. The decision curve showed that using the combined model to predict HIE added more benefits than using the clinical and radiomic models.

### 3.7. Reproducibility

In radiomic feature extraction, the ICC for inter-observer reproducibility was satisfactory. The ICC values of the features extracted by observers 1 and 2 during their first extraction ranged from 0.785 to 0.892.

## 4. Discussion

Hypoxic-ischemic encephalopathy is typically diagnosed based on clinical presentation, including Apgar score and paired cord blood gas. Paired cord blood gas can objectively reflect the degree of fetal hypoxia and is the objective basis for reflecting fetal intrauterine hypoxia. However, paired cord blood gas examinations are not comprehensively performed in in China. Furthermore, the Apgar scores are highly subjective. MRI is used for the assessment of severity and outcomes. The use of MRI to identify and define HIE within a short time is particularly challenging because of the different examination times, injury patterns, and protocols. Furthermore, some patients can have normal or pseudo-normal MRI findings.

The cerebral venous system consists of two parts: The superficial cerebral venous system and the deep cerebral venous system. The superficial cerebral venous system consists primarily of the cerebral cortical and subcortical veins, including the superficial cerebral veins, pial veins, cortical veins, and venous sinuses. The deep cerebral venous system mainly consists of veins of the deep brain medulla, basal nucleus, internal capsule, diencephalon, and ventricular choroid plexus in the cerebral hemisphere (Mankad et al., 2019). A typical fan-like pattern can be seen in the DMVs, which drain blood from the WM to the subependymal veins (Filip et al., 2022). SWI was used to determine the difference in magnetic sensitivity between the different tissues to form an image comparison and a thin-layer reconstructed gradient-echo imaging sequence with the three-dimensional acquisition, completed flow compensation signal, and high signal-to-noise ratio. Due to their smaller size on SWI, DMVs are better seen when there is increased intravenous deoxyhemoglobin, venous congestion, or thrombosis, but not in the MR venography sequences in neonates (Khalatbari et al., 2020). Compared with conventional MRI sequences, SWI is superior in delineating cerebral venous structures in both healthy subjects and patients with HIE. Therefore, it is easier to display the DMV area on SWI to obtain the ROI more accurately.

There are relatively few studies on neonatal DMVs. DMV engorgement seen in neonates is thought to be a type of perinatal venous stroke (Khalatbari et al., 2020). Severe WM injury in preterm infants may be caused by DMV congestion or thrombosis, which can later develop into periventricular leukomalacia (Chen et al., 2020). A scoring system for MR was developed by Kitamura et al. (2011) to predict neurologic outcomes when DMV congestion and thrombosis are present. The first application of texture to SWI in infants was performed by Kim et al. (2020) who showed significant differences in the DMVs between preterm infants and term infants (Thayyil et al., 2010). Therefore, SWI may be more sensitive in detecting abnormalities when the neonatal brain tissues are hypoxic and have a developmental correlation.

In this study, we used manual segmentation to extract and select image radiomic features with good predictive values by outlining the ROI of neonatal SWI images. First, we developed and validated a radiomic model for an accurate diagnosis of HIE in neonates. The AUC of the radiomic signatures in the validation cohort was 0.93 [95% confidence interval (CI), 0.733–0.905], and the accuracy, sensitivity, and specificity were 0.831, 0.807, and 0.885, respectively. In the training cohort, the AUC of the radiomic signatures was 0.94 (95% CI, 0.829–0.924), and the accuracy, sensitivity, and specificity were 0.883, 0.857, and 0.936, respectively. Based on the SWI features, the radiomic model of the DMVs has a higher prediction efficiency for neonatal HIE than clinical model. We found that the Rad-score, sex, ALT, urea nitrogen, and pH were important indicators for differentiating between patients with HIE and controls, and that other clinical features were not potential predictors. Then, based on the independent predictors of clinical characteristics, combined with the Rad-score, we developed and validated a nomogram. The AUCs in the training and verification cohorts were 0.97 and 0.95, respectively. Compared with the clinical model (AUC, training: 0.86, validation: 0.85) and radiomic models (AUC, training: 0.94, validation: 0.93), this model demonstrated a superior predictive effect. A significant difference was found between the ROCs of the radiomic model and the combined model using the Delong test, which showed that the addition of clinical features can significantly improve the predictive value of the radiomic model in distinguishing the HIE group from the normal group. The DCA results showed that the combined model was more effective in predicting neonatal HIE than both radiomic and clinical models. Our analyses demonstrated that the Rad-score, gestational age and ALT were important indicators for differentiating between patients with HIE with good outcomes and those with poor outcomes. Based on the independent predictors of clinical characteristics, combined with the Rad-score, we developed a combined nomogram model. The AUCs in the training and verification cohorts were 0.91 and 0.88, respectively. The DCA results showed that the combined nomogram model was more effective in predicting the neurologic outcomes after hypoxic-ischemic injuries than both the radiomic and clinical models. Thus, there is clinical value in assessing the radiomic features of DMVs.

Venule dilation can be observed in SWI images, but conventional MRI provides limited information based on human perception alone. In our study, 38 patients showed completely normal MRI findings. In contrast, 65 of 152 patients showed DMV engorgement on SWI. We first added a clinically independent factor combined with radiomic features to establish a nomogram model. The nomogram model visualizes the radiomic features and clinical predictors and provides a simple and easy-to-use tool for individualized evaluation of HIE in neonates.

Textural features can be used to quantitatively differentiate infants with ischemic injuries. Kim et al. (2020) only used a first-order histogram analysis to assess feasibility in infants with ischemic injury. For very few infants, the AUC was 0.865, which was lower than that in our study. Sarioglu et al. (2022) demonstrated an accurate diagnosis of moderate-to-severe HIE in neonates based on the texture of the basal ganglia and thalamus. Sensitivity and accuracy were 95 and 94.3%, respectively. In this model, only textural features were used. While textural features are very useful for identifying target images with obvious texture features, their main disadvantage is that when the resolution of the image and the illumination of the target change, the texture of the target image may produce a large deviation and affect the classification effect. Furthermore, our study included 279 patients, as opposed to the 7–35 patients in previous studies. Studies with small sample sizes can lead to overfitting and affect the authenticity of the data.

While it remains unclear how radiomic and nomogram models reflect the damage found in HIE, we speculate that radiomics and nomograms can reveal micro-changes in hypoxic injuries. Gas exchange disturbances occur in neonatal HIE. Early pathological changes in HIE mainly include nerve cell degeneration, necrosis, brain edema, intracranial hemorrhage, and cerebellar injury. In the late stages, encephalomalacia and brain atrophy may occur. After hypoxia of the brain tissues, cerebral blood flow perfusion decreases, arterioles show reactive dilation. In order to maintain oxygen metabolism, cerebral blood flow increases. Hemodynamics at the level of cerebrovascular histology shows compensatory damage, which increases the proportion of deoxyhemoglobin in venules (Shankaran et al., 2012; Goergen et al., 2013). SWI showed that the sensitivity of intracranial savings and abnormal venous dilatation in the brains of neonates with HIE was higher than that of routine MRI sequences, which accurately evaluated the damage in HIE (Kitamura et al., 2011).

A series of quantitative imaging features can be extracted from SWI-based DMVs. Wavelet_HHL_gldm_DependenceVariance and wavelet_HLH_glcm_lmc1 were considered as key parameters for the accuracy of the proposed SWI model according to their corresponding coefficients. The GLDM quantifies gray-level dependencies in an image. Gray-level dependency is defined as the number of connected voxels within a distance δ that is dependent on the center voxel. The gray-level band matrix includes features that describe the distribution of small/large areas and low/high gray areas. The GLCM is a matrix whose row and column number gray values and cell contain the number of times the gray value is in a certain relationship (angle and distance), also known as a second-order histogram. The features calculated using the GLCM included entropy, energy, contrast, homogeneity, dissimilarity, and correlation. Although it is not possible to interpret precisely, we can infer that a larger value of GLDM and GLCM may indicate a stronger distribution of high gray areas and gray values, since higher results than the threshold value elucidate the HIE, according to our findings. In addition to predicting neurologic outcomes after hypoxic-ischemic injuries, the log_sigma_2.0_mm_3D_first-order_90th percentile and wavelet_HLL_first-order_mean were considered as key parameters. These characteristics reflect the symmetry, uniformity, and local intensity distribution changes of the measured voxels. Statistically, the characteristics of the gray value of the image can be calculated. We found those first-order parameters were useful for discriminating between good and poor neurologic outcomes.

This study has several limitations. First, as this was a single-center retrospective study, external verification was not possible. As a result, case selection bias may have occurred, and generalizability may be limited. Second, manual segmentation was used to delineate the ROI of the DMVs. However, automatic or semi-automatic segmentation was not used for comparison and verification. This may have had a subjective impact. Third, because hypothermia treatment was routine for severe HIE and thus, could affect the radiological images and metabolite values on MRI, we did not exclude neonates treated with hypothermia. Fourth, further studies are needed to confirm that this subject can be used to develop a model showing differences in the degrees and stages of ischemia. Finally, the timing of imaging, which may affect the outcome of HIE patients, may also have an impact on the radiomics. Therefore, further studies with different scan times are needed to validate our results. These deficiencies need to be addressed in the future.

## 5. Conclusion

A combined nomogram model incorporating Rad-scores and independent clinical factors, as well as a radiomic model, can be a reliable and effective tool for evaluating HIE. Despite not being visibly detectable, objective features may indicate differences in the DMVs on SWI. Considering the results of this study, we suggest that the radiomic analysis of SWI can be a useful tool for identifying infants with HIE. It also laid a foundation for predicting the stage and prognosis of HIE by using radiomics-based of DMWs of SWI.

## Data availability statement

The original contributions presented in this study are included in the article/Supplementary material, further inquiries can be directed to the corresponding author.

## Ethics statement

The studies involving human participants were reviewed and approved by the medical ethics committee of the Hunan Children’s Hospital of South China University. Written informed consent from the participants’ legal guardian/next of kin was not required to participate in this study in accordance with the national legislation and the institutional requirements.

## Author contributions

KJ and XZ designed the study. YY and XZ collected the data. HL performed the statistical analysis. JL and XZ drafted the manuscript. KJ revised the manuscript. All authors read and approved the final manuscript.

## Footnotes

1
https://www.ibm.com

2
https://www.Rproject.org
