# Supplementary material for: A radiomics-based study of deep medullary veins in infants: Evaluation of neonatal brain injury with hypoxic-ischemic encephalopathy via susceptibility-weighted imaging
Source: Front Neurosci. 2023 Jan 17;16:1093499. doi: 10.3389/fnins.2022.1093499 (PMC9887113; doi:10.3389/fnins.2022.1093499)
Supplement: Supplementary file 1 [file Data_Sheet_1.docx]

**Supplementary Files**

**MRI imaging acquisition**

All brain MRI scans were performed in our hospital on 3.0 T MRI scanner (MAGNETOM Skyra, Siemens or MAGNETOM Prisma, Siemens.) with an eight-channel head coil using the same MR parameters. Axial SWI was used for extraction of radiomics features. The parameters for SWI were field-of-view 200$\times$90.6 mm; voxel size 0.8$\times$0.8$\times$2.0 mm; slice thickness 2.0 mm; TR 27.0 ms; TE 20.0 ms. The MR images of enrolled patients were exported in Digital Imaging and Communication in Medicine (DICOM) format in institutional picture archiving and communication system (PACS), and then converted to the NIFTI format using AK software (Artificial Intelligence Kit v.3.1.0.A, GE Healthcare).

**Image Preprocessing**

We used AK software (Artificial Intelligence Kit v.3.1.0.A, GE Healthcare) to perform this procedure. The process included: First, to eliminate the intrinsic dependency on voxel size for the radiomic features, a resampling method with a linear interpolation algorithm was used to normalize the voxel resolution. Higher-order texture analysis features were derived from different directions and different scales, so the anisotropic voxels scanned at other sizes were resampled to form voxels, i.e, 0.750 mm* 0.750 mm* 0.750 mm. Second, a Gaussian filter was used to remove “unwanted signals”. Because different scanners had the same gray level, gray level normalization was not performed here.

**Supplementary Figure 1** SWI with ROI placement


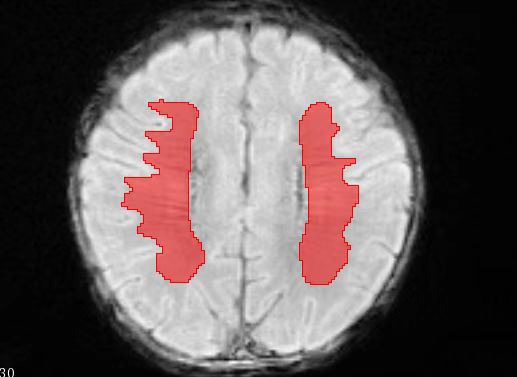


**Supplementary Figure 2** The least absolute shrinkage and selection operator (LASSO) including the selection of the regular parameter \(\lambda\) and determination of the number of features.


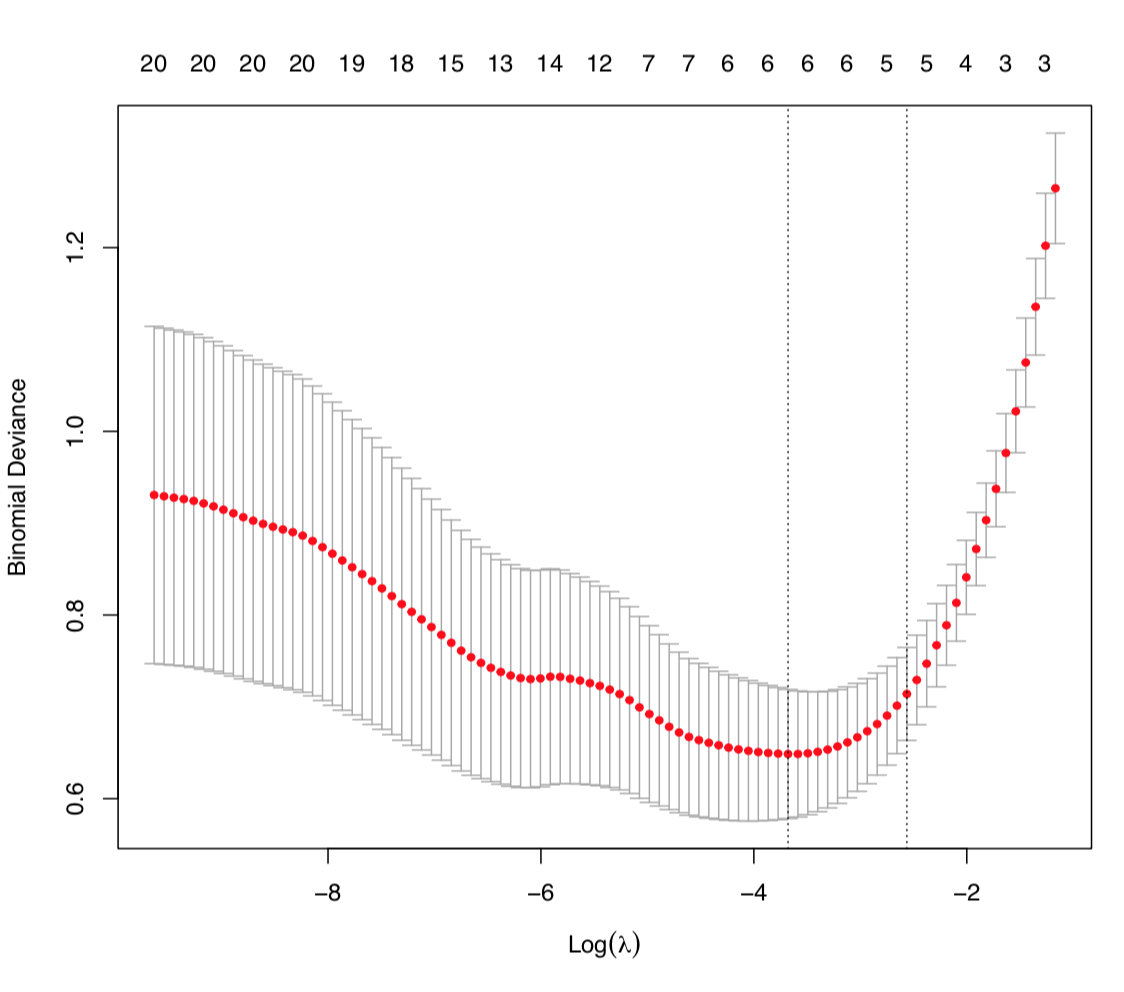


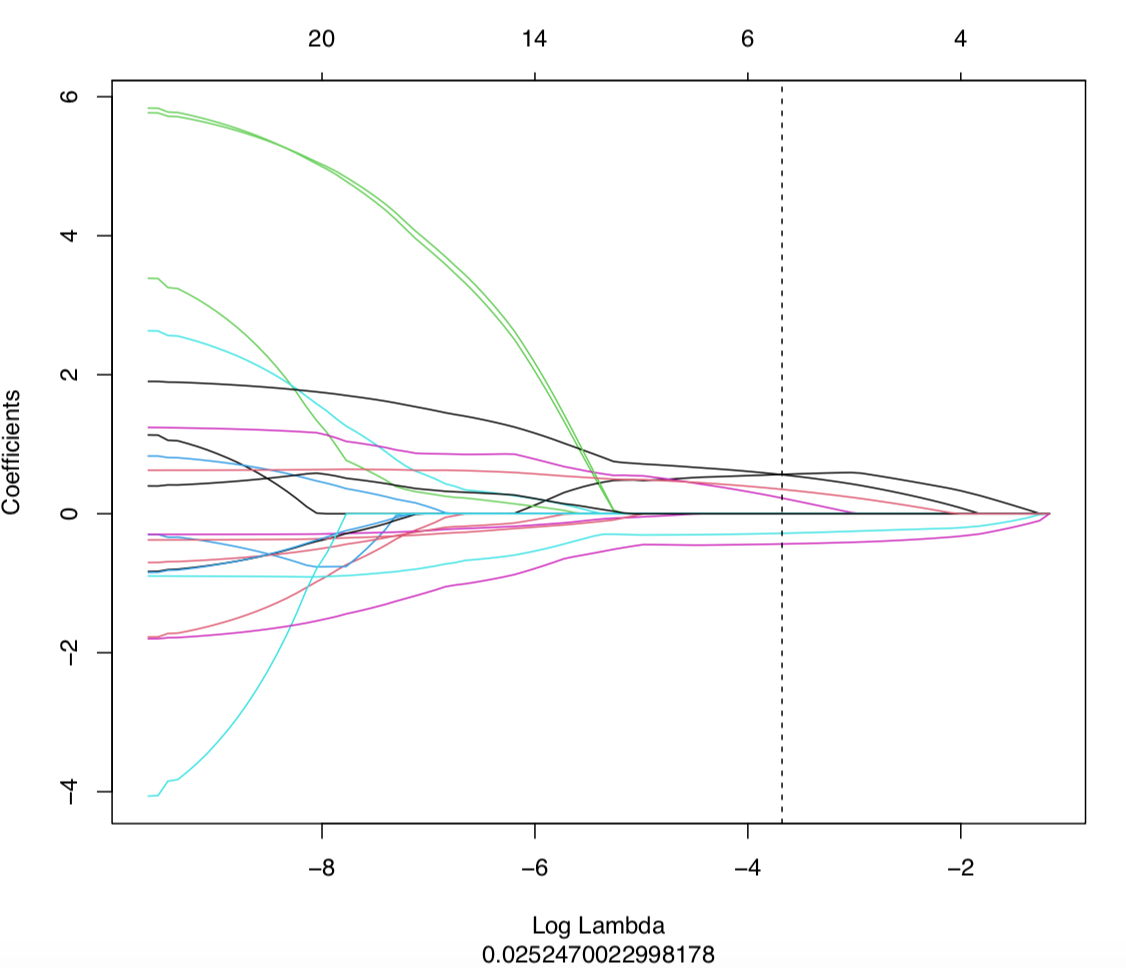


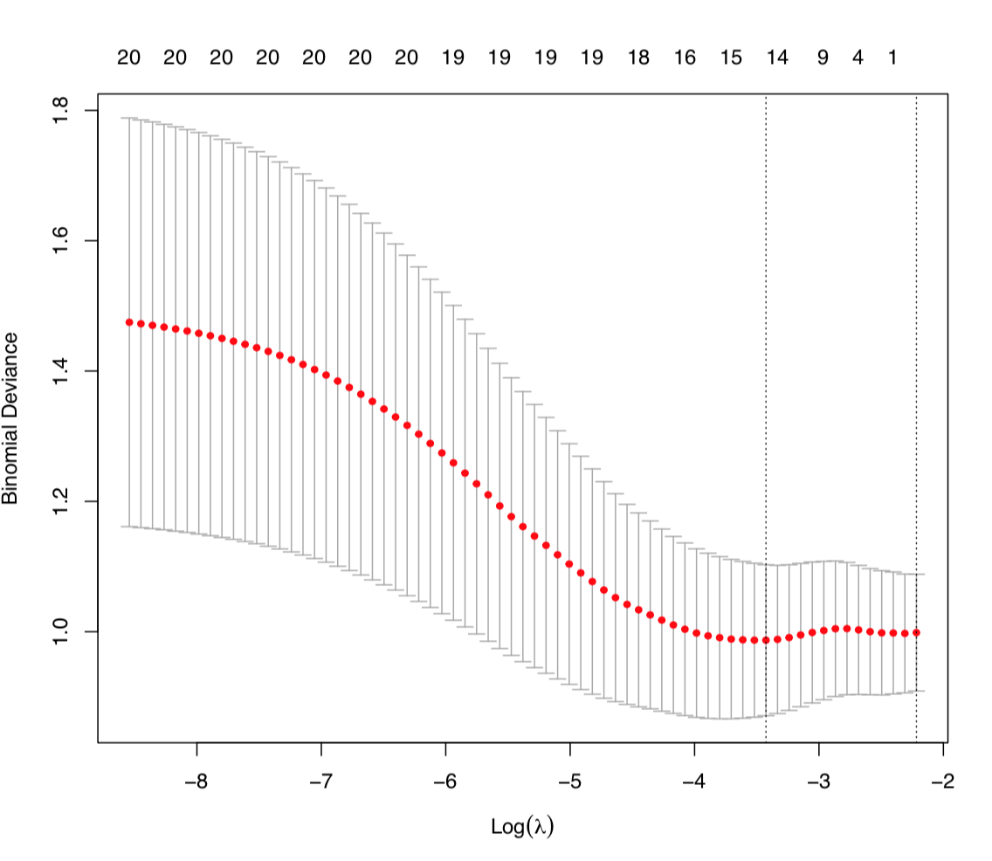


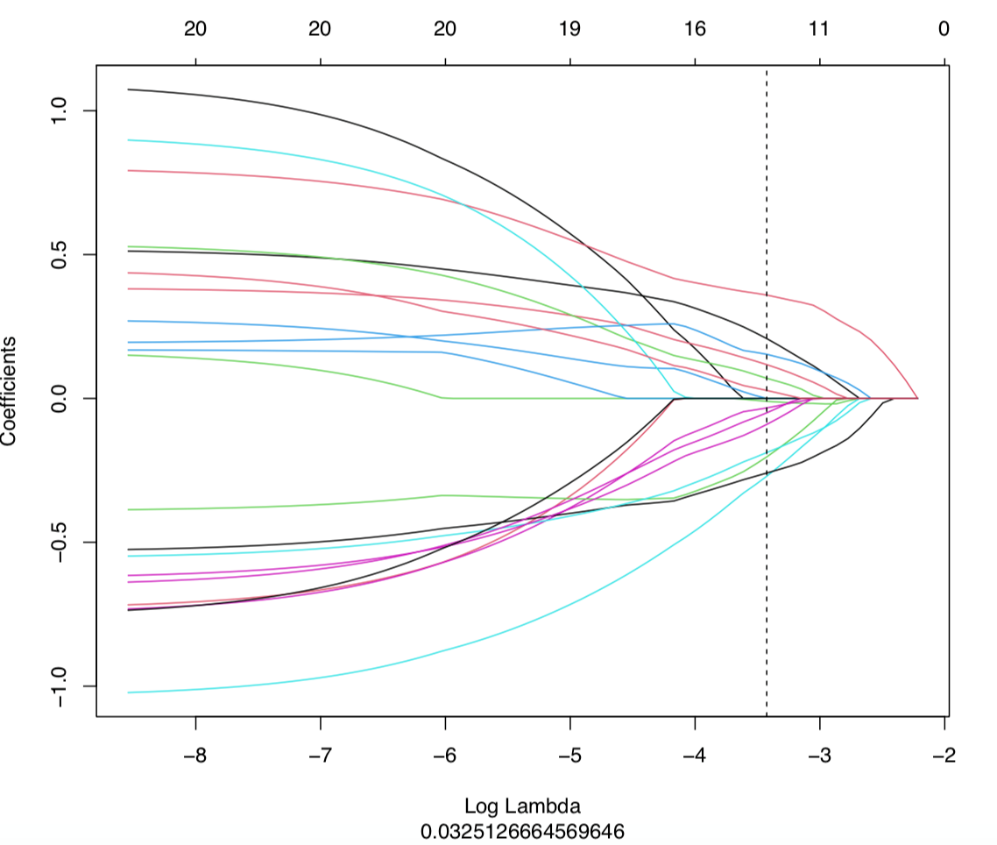


**Supplementary Figure 3** A Selection of the most predictive subset of features with the corresponding coefficients after the number of features was determined. B Demonstration of significantly higher Rad-scores in the HIE group (Label=1) than those of the normal group (Label=0), in both the training cohort and the validation cohort.

A B


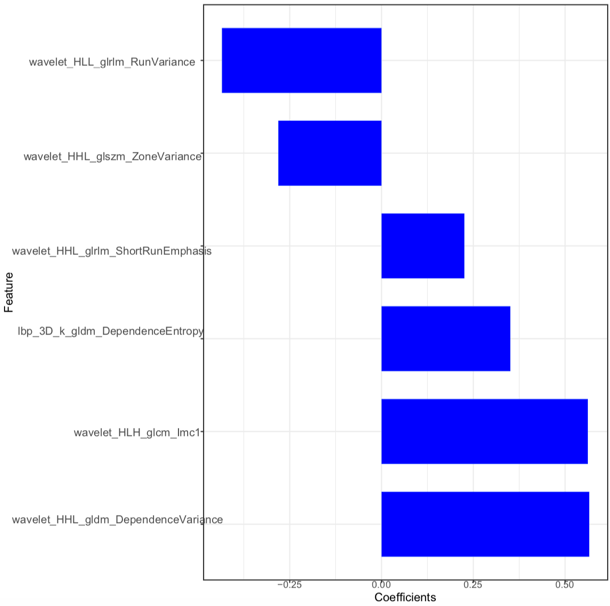

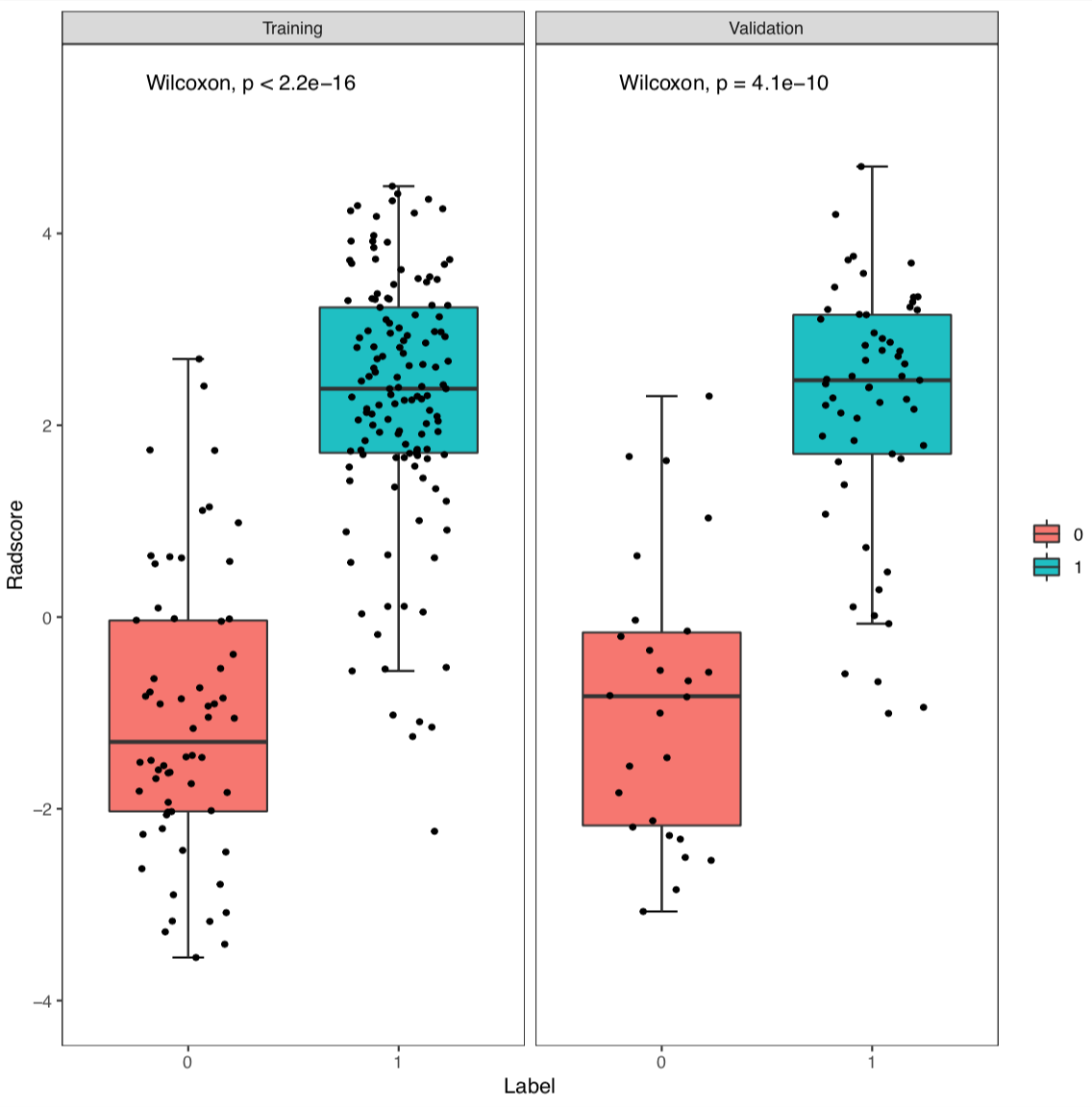


**Supplementary Figure 4** A Selection of the most predictive subset of features with the corresponding coefficients after the number of features was determined. B Demonstration of significantly higher Rad-scores in the poor outcome group (Label=1) than those of the good outcome group (Label=0), in both the training cohort and the validation cohort.

A B


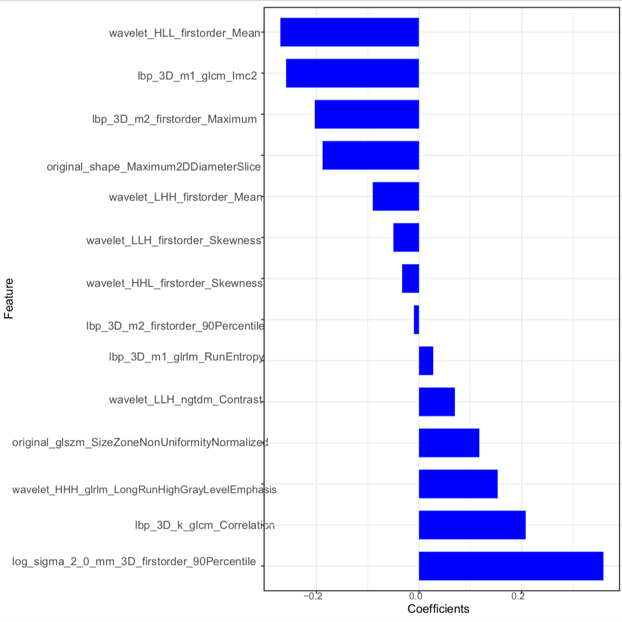

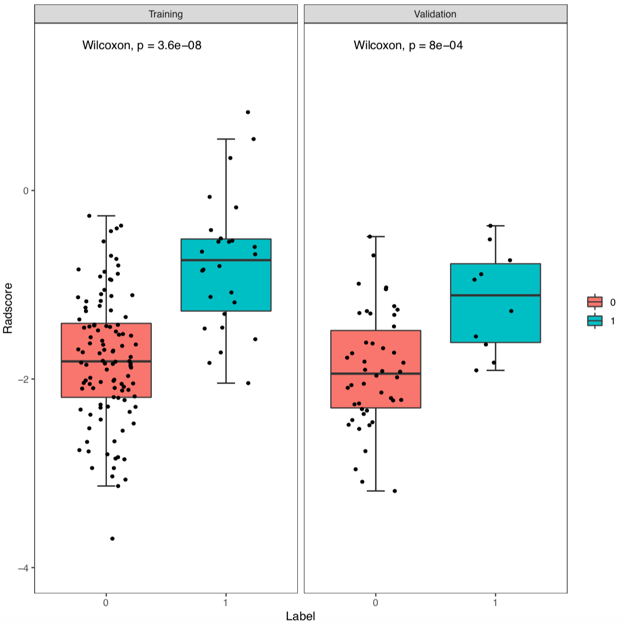


**Supplementary Figure 5** Calibration curves (HIE vs normal) of the three models for the training cohort (A) and validation cohort (B).

A B


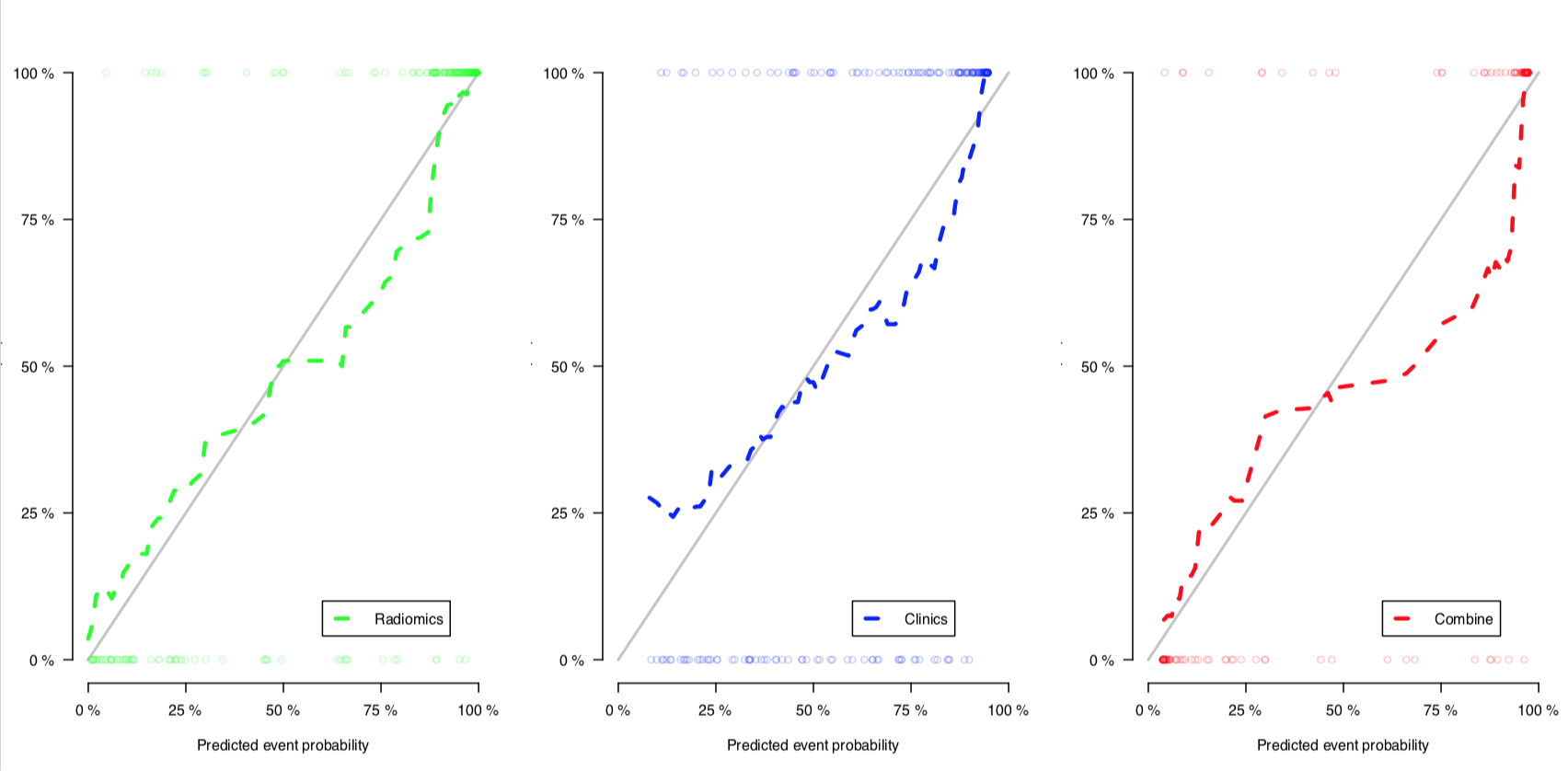

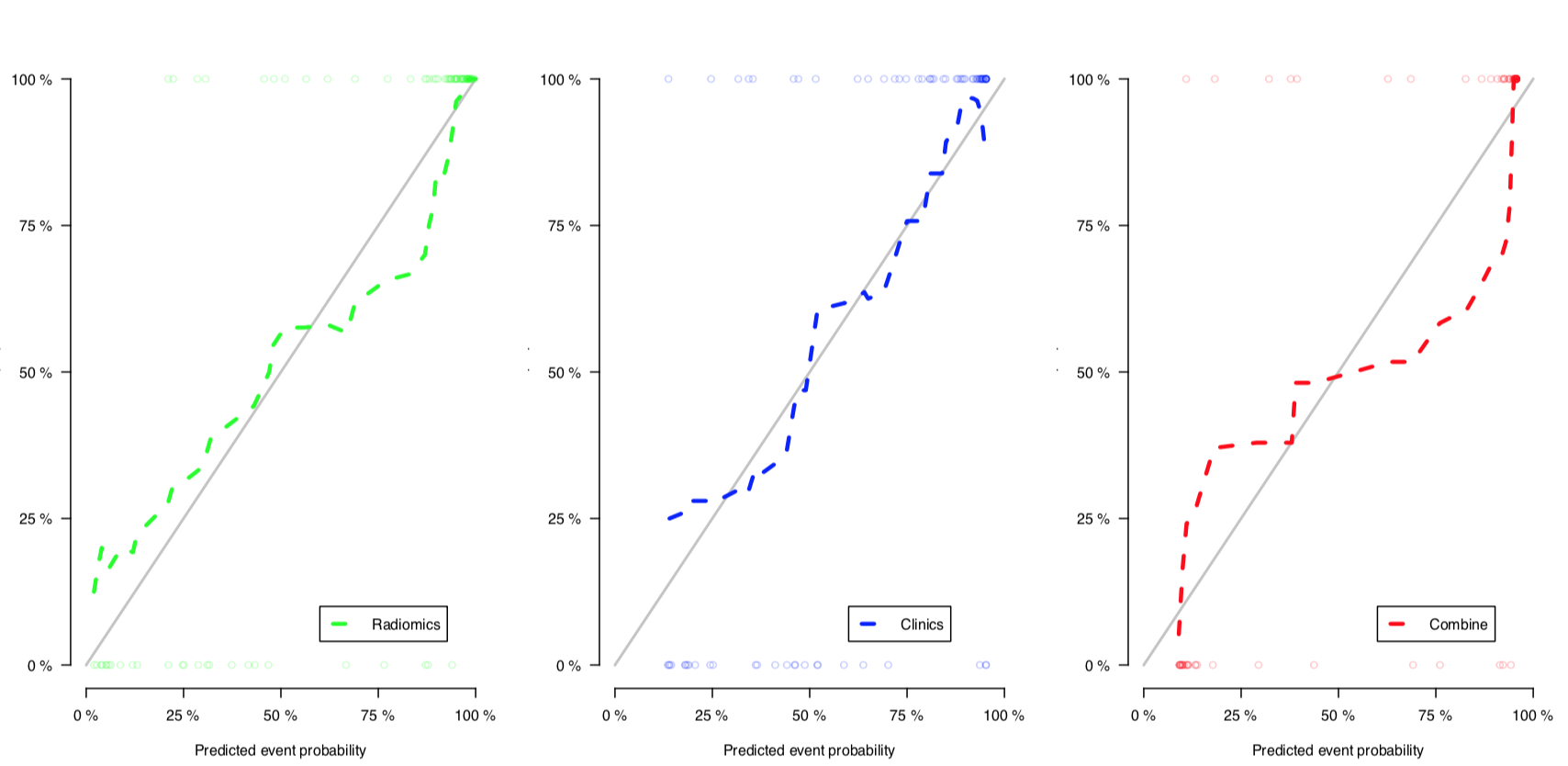


**Supplementary Figure 6** Calibration curves (good outcome vs poor outcome) of the three models for the training cohort (A) and validation cohort (B).

A B


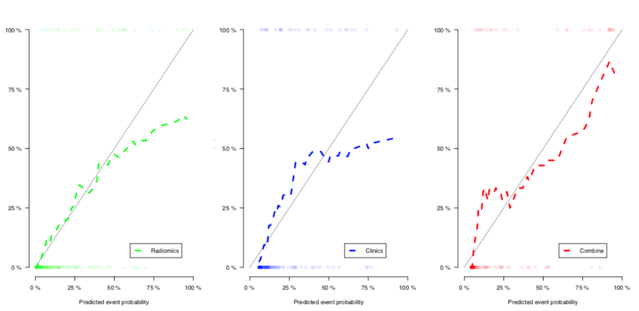

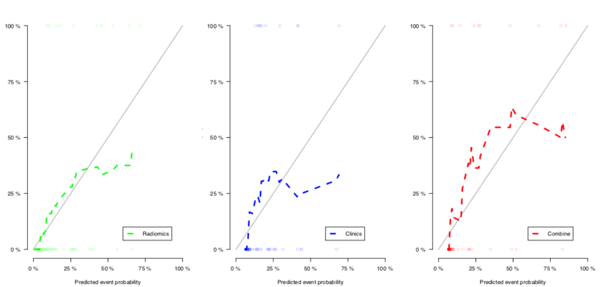


**Equations**

**Equation 1**

Rad-score formula

"radscore=1.32006780702773*(Intercept)+0.565766692758406*wavelet_HHL_gldm_DependenceVariance+0.562005515683456*wavelet_HLH_glcm_Imc1+0.351059222506586*lbp_3D_k_gldm_DependenceEntropy+0.225518910861267*wavelet_HHL_glrlm_ShortRunEmphasis+-0.281130196431422*wavelet_HHL_glszm_ZoneVariance+-0.434651285620066*wavelet_HLL_glrlm_RunVariance"

**Equation 2**

> print(radscore)

[1] "radscore=0.359326886646572*log_sigma_2_0_mm_3D_firstorder_90Percentile+0.207997061524047*lbp_3D_k_glcm_Correlation+0.153447521399667*wavelet_HHH_glrlm_LongRunHighGrayLevelEmphasis+0.117544477300047*original_glszm_SizeZoneNonUniformityNormalized+0.0700859285298625*wavelet_LLH_ngtdm_Contrast+0.027832584868765*lbp_3D_m1_glrlm_RunEntropy+-0.00996333147822123*lbp_3D_m2_firstorder_90Percentile+-0.0330449075209615*wavelet_HHL_firstorder_Skewness+-0.0499605184455783*wavelet_LLH_firstorder_Skewness+-0.0902811546502284*wavelet_LHH_firstorder_Mean+-0.188063895199925*original_shape_Maximum2DDiameterSlice+-0.203170074230495*lbp_3D_m2_firstorder_Maximum+-0.259149358768634*lbp_3D_m1_glcm_Imc2+-0.270425302339894*wavelet_HLL_firstorder_Mean+-1.59612780147925*(Intercept)"

> sink()

**Equation 3**

Nomocore formula

[1] "nomoscore=30.801197443253*(Intercept)+-1.48168022511483*Gender+0.114126809172124*ALT+0.425134281755873*Urea_nitrogen+-4.48771148922369*PH+1.35655230019758*Radscore"

**Equation 4**

> print(nomoscore)

[1] "nomoscore=-40.811082677789*(Intercept)+1.02932420369325*Gestational_age+1.90795712172445*Convulsions+0.0083307886574285*ALT+2.44654677908888*Radscore"

> sink()
